# Supplementary material for: NET Proteome in Established Type 1 Diabetes Is Enriched in Metabolic Proteins
Source: Cells. 2023 May 5;12(9):1319. doi: 10.3390/cells12091319 (PMC10177393; doi:10.3390/cells12091319)
Supplement: Supplementary file 1 [file cells-12-01319-s001.zip › Supplementary figures - Neutrophils and type 1 diabetes - Cells.pdf]

SUPPLEMENTARY FIGURES

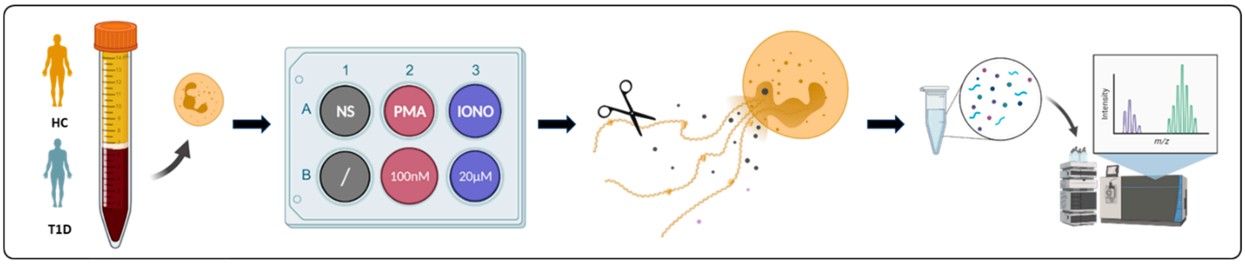

Supplementary Figure S1: NET protein harvesting workflow for proteomic analysis. Scheme overview of the workflow from blood sampling, NET induction and NET protein harvesting to loading and analysis by LC-MS/MS (created with Biorender.com). Abbreviations: HC: healthy control, T1D: type 1 diabetes, NS: unstimulated, PMA: phorbol-myristate acetate, IONO: ionomycin.

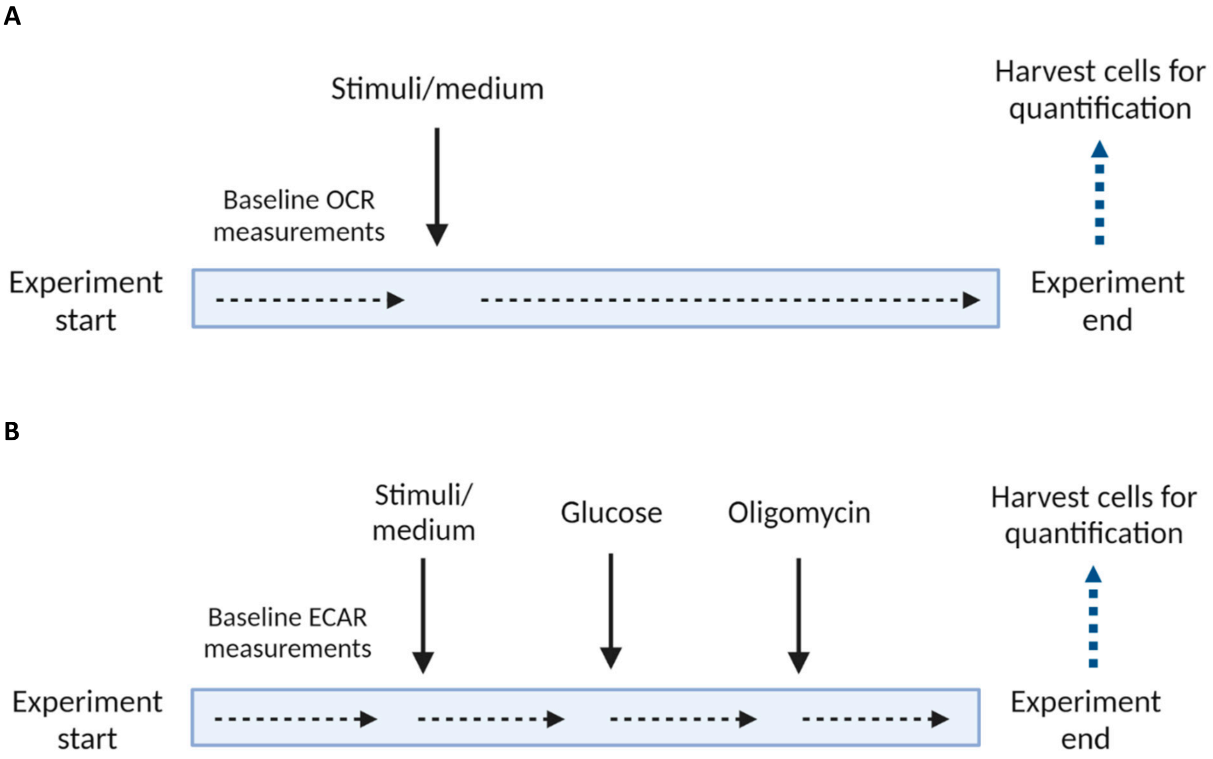

Supplementary Figure S2: Bioenergetic profile of HC and T1D neutrophils in response to PMA and ionomycin. (A, B) Scheme overview of Seahorse extracellular flux analysis of mitochondrial respiration (OCR, **A**) and extracellular acidification (ECAR, **B**) of HC and T1D neutrophils, in resting and PMA- or ionomycin-stimulated conditions (create with Biorender.com). Black arrows indicate timepoints at which the stimuli and compounds were injected.

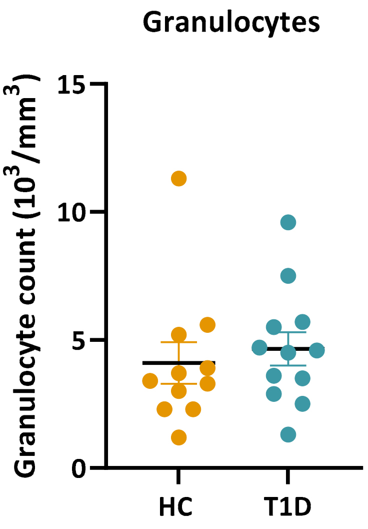

Supplementary Figure S3: Granulocyte counts in peripheral blood of HC and long-standing T1D subjects. Reported as $10^3/mm^3$ of total blood. Unpaired Mann-Whitney test.
